# Levels of Competence and Need for Continuing Education in Nonspecialist Palliative Care Settings—A Qualitative Study of Views from Finnish Health Care Professionals

**DOI:** 10.1089/pmr.2024.0060

**Published:** 2024-12-23

**Authors:** Hanna Hävölä, Anu Helmikkala, Anu Viitala, Elina Kiviniemi, Pauli Lamppu, Heidi Keronen, Minna Hökkä

**Affiliations:** ^1^ Pirkanmaa Hospice, Tampere, Finland.; ^2^ Department of Nursing Science, University of Eastern Finland, Kuopio, Finland.; ^3^ Wellbeing Services County of Pirkanmaa, Tampere, Finland.; ^4^ Tampere University, Tampere, Finland.; ^5^ Wellbeing Services County, Central Uusimaa, Finland.; ^6^ Department of general practice and primary health care, University of Helsinki, Helsinki, Finland.; ^7^ Wellbeing Services County of Kainuu, Kajaani, Finland.; ^8^ Wellbeing Services County of North Ostrobothnia, Oulu, Finland.; ^9^ Diaconia University of Applied Sciences, Oulu, Finland.

**Keywords:** competencies, education, nursing, palliative care, professional competence, qualitative research

## Abstract

**Background:**

The need for palliative care (PC) is increasing due to the growing number of chronic diseases and an aging population. As such, the requirement to ensure the provision of PC is evident. This calls for PC competence for nurses working in nonspecialist PC settings.

**Objective:**

The aim was to describe the views of Finnish health care professionals relating to PC competencies and the development needs for continuing education in nonspecialist PC settings.

**Design:**

A qualitative study design.

**Setting/subjects:**

The data were compiled via an e-survey from health care professionals working in nonspecialist PC settings in Finland; 281 participants answered the open question: “Tell us what you think about the competencies in palliative care.”

**Measurements:**

The data were analyzed using inductive content analysis.

**Results:**

The description of PC competence was categorized into four main categories, including 64 subcategories. The main category containing the largest number of reduced expressions (*f* = 303) was “Perceived level of PC competence and development needs.” The competence in PC was also identified as “Perceived need for continuing education in different palliative care competencies” (*f* = 243), “Building the foundations of one’s own competence” (*f* = 133), and “Factors related to the work organization and connected to the competence enhancement” (*f* = 84).

**Conclusion:**

The health care professionals in nonspecialist PC settings recognize the importance of ensuring competence and the need for continuous and regular education. The results of this study can be utilized in the planning of continuing education and in targeting it correctly.

## Introduction 

Palliative care (PC) aims to alleviate suffering and improve the quality of life of patients and their families when they face problems related to life threatening illnesses. The PC approach is holistic, taking into account the physical, psychological, social, or spiritual dimensions of challenges which the families and patients face.^1^ The need for PC is increasing rapidly due to an aging population and the growing prevalence of noncommunicable diseases.^2,3^ The burden of serious health-related suffering is expected to almost double by 2060, with the fastest increases occurring among older people and those with dementia.^4^ Despite the fact that both the World Health Organization and the Council of Europe emphasize that access to PC should be considered a human right^5,6^ and inequities have been identified as health care priorities, injustice regarding access to PC and pain relief has still been largely ignored.^7^ PC should be integrated within health care and provided at all levels of care. Early identification of patients with PC needs should be carried out in primary care services and much of the care of dying patients should be provided by primary care professionals.^2^

To successfully provide PC, all health care professionals need to have sufficient competence in the subject.^3^ Insufficient PC education has been identified as a major barrier to the development of PC.^8^ Therefore, one of the keys to reducing inequalities in PC is to develop PC competence and education. Nurses have an important identified role in both the provision of PC and enabling access to it,^9^ and they are the largest professional group when representing 59% of all health care groups.^10^ Nurses spend the most time with the patient and their closest ones. Therefore, nurses have an important role in terms of coordinating care, ensuring the continuity and quality of care, and supporting the patient and their family.^11,12^ To achieve these demands, it is essential that nurses have appropriate PC competence. Competencies in this study were defined as knowledge, skills, values, and attitudes (heightened through self-awareness) that a nurse should possess to successfully perform quality PC.^13,14^ To ensure these competencies, all nurses should have access to PC education at the undergraduate level.^15,16^ In addition, continuing education programs in practice settings for nurses working with patients with PC needs should be enabled.^15,16^

It is known from previous research that unpreparedness when caring for patients with palliative and end-of life care issues increases professionals’ experiences of high levels of exhaustion, burnout, illness, and moral distress,^17,18^ as well as vicarious trauma, which has been seen especially during the Covid 19 pandemia.^19^ Nevertheless, nurses working in different settings still report suboptimal knowledge regarding PC.^20,21^ However, PC education has the potential to increase nurses’ perceived self-efficacy,^22^ and education interventions have positive effects on PC knowledge and positively influences nurses’ attitudes toward end-of-life care.^23^ In Finland, inequality regarding access to PC services within the country and a lack of PC competence and education were highlighted in a report published by the Ministry of Social Affairs and Health.^24^ The FinPall-project, funded by the Ministry of Social Affairs and Health, was subsequently launched to improve these identified gaps. The aim of the project was to support the development of PC services and to strengthen competency, especially in nonspecialist PC settings. This study is part of the FinPall-project.

Education, and appropriate competence, can promote the well-being of nurses and, most importantly, promote better PC for patients in nonspecialist PC settings. To improve continuing education and competencies, it is essential to explore what needs to be developed in nonspecialist PC environments.

## Methods

The purpose of this qualitative study was to describe the views of Finnish health care professionals relating to PC competencies and the development needs for continuing education in nonspecialist PC settings. To present a comprehensive summary of the phenomenon of interest, a descriptive qualitative study design was applied.^25^

### Data collection and sample

The data were compiled through a web-based survey as part of a larger national cross-sectional survey that targeted health care professionals working in nonspecialist PC settings in Finland. The survey was carried out online from November to December 2022, using the Webropol survey tool. The survey response link was shared via social media (Facebook). The project network was then informed of the launch of the survey and advised where the survey could be found. The survey (Supplementary Data S1) included 63 quantitative self-evaluations items on different competencies, which will be reported in another article. The survey included one open-ended question: “Tell us about your thoughts on palliative or end-of-life care competence.” The content validity was pretested by a group of health care professionals and no need for revision was identified. The pretest responses were not included in the analysis.

Of the 662 eligible answers, 281 answers were given to the open-ended question, the answers to which are included in this study. The majority of respondents were nurses, but some nursing students, physicians, elderly care professionals, public health nurses, physiotherapists. and care managers who did not have a nursing background also responded and were included. Participant characteristics are shown in Table 1.

**Table 1.** Participant Characteristics of Health Care Professionals (*n* = 281)

Characteristics	%	*n*
Nonspecialist PC setting		
Nursing home	42	118
Hospital ward	30	84
Home care	15	42
Hospital-at-home	4	12
Other	9	24
Educational background		
Practical nurse	37	103
Registered nurse	44	124
Master’s degree or higher	11	31
Other	8	23
Receiving ongoing training in PC		
Regularly or enough	40	111
Not enough	45	126
Not at all	15	41
Mainly working		
Clinical work/bedside	80	225
Supervisor	16	46
Other	4	10
Working experience within palliative care		
>15 years	27	73
11–15 years	17	46
6–10 years	26	68
2–5 years	16	42
<2 years	10	26
No experience	4	12
Caring for PC patients		
Daily	30	84
Weekly	21	59
Monthly	19	53
Less frequently or not at all	30	84

PC, palliative care.

Participation in the study was voluntary, and responses were given anonymously. Information about the purpose of the survey was given, and informed consent was obtained before respondents were able to proceed with the survey. The standards of the Declaration of Helsinki were followed during the whole study.^26^ Since the participants’ integrity was not compromised in this study, a formal ethical approval was not needed according to Finnish law.^27^

The research questions in this study are:

1. What are the health care professionals’ views of palliative care competency in nonspecialist settings?

2. What are the development needs for continuous palliative care education in nonspecialist settings?

### Data analysis

The answers to the open question in the survey material were first separated into their own document. The qualitative analysis was performed by an inductive content analysis, in which categories emerge from the data.^28^ The manifest content of the material was the focus of the analysis. The inductive content analysis was performed via three phases: The first phase included reducing meaningful expressions answering the research questions. In the second phase, the similar reduced expressions were clustered to subcategories, and in the third phase, the categories and main categories were formed.^28^

At the beginning of the reduction phase, three researchers (HH, AH, and MH) discussed and agreed that the unit of analysis would be a word, phrase, or sentence. Two researchers (HH and AH) performed the reduction phase by reading through the material while asking the research questions. Coding was done manually using colors and a matrix. An example of the analysis process is presented in Table 2. The number of reduced expressions was counted to present the frequencies (*f*) for each category. The total number of reduced expressions was 763.

**Table 2.** Example of the Analysis Procedure, Palliative Care Networks Strengthening Competence

Meaningful expressions	Reduced expressions	Subcategory
The central hospital’s palliative network organizes training regionally once a year and these trainings have been perceived as good. (P231)	The regional trainings of the central hospital’s palliative network once a year has been perceived as good. (P231)	Palliative care networks strengthening competence (*f* = 4)
Palliative care expertise is improved by the network of palliative care nurses started in the region. (P231)	The network of palliative care nurses improves competence in the region. (P231)	
Competence varies from place to place; networking has brought further positive structures to this. (P648)	Networking has brought positive structures to competence. (P648)	
We have received good practices from each other, etc. (P648)	Networking has made it possible to share good practices. (P648)	

During the reduction phase, it was noticed that the participants had described competence more broadly, for example, in their assessment of the level of competence and definition of the factors that they thought were the basis of competence. These expressions were also included in the analysis.

The reduced expressions were clustered and later categorized by the first author (HH), and the results were checked and discussed by the other authors (AH and MH). Moreover, all the authors commented on the results.^28^ During the analysis, data saturation was achieved, which meant that no further data collection was necessary.^29^

### Findings

The views of health care professionals regarding PC competencies and development needs for continuous PC education included 4 main categories, 17 categories, and 64 subcategories, which are presented in Tables 3 and 4. The main categories, categories, and two subcategories containing the most reduced expressions are presented further in the findings. The original expressions from the data are presented in italics in the findings.

**Table 3.** Health Care Professionals’ Views of PC Competency in Nonspecialist Settings

Main category	Category	Subcategory
Building the foundations of one’s own competence (*f* = 133)	The competence developed from work experience and support from a more experienced colleague (*f* = 6*5*)	Competencies growing from work experience (*f* = 30) The lack of PC competencies due to minor work experience (*f* = 13)
		The importance of the support from a more experienced colleague in ensuring competence (*f* = 11)
		The importance of orientation in ensuring competence (*f* = 6)
		The support doctors need in their clinical work (*f* = 5)
	Support from the specialist level of PC (*f* = 51)	PC specialist strengthening competence (*f* = 44)
		PC networks strengthening competence (*f* = 4)
		Palliative center strengthening competence (*f* = 3)
	Education as the foundation of PC competence (*f* = 17)	Postgraduate education as the foundation of competence (*f* = 13)
		Undergraduate education as the foundation of competence (*f* = 4)
Factors related to the work organization and connection to competence enhancement (*f* = 84)	Employer-related obstacles for achieving competencies (*f* = 47)	The lack of continuing education provided by the employer (*f* = 21) The connection of rarely occurring PC encounters to competence (*f* = 12)
		The lack of resources hindering competence enhancement (*f* = 7)
		The high cost of continuing education (*f* = 4)
		The need for unifying nursing skill requirements within PC (*f* = 3)
	Employer-related factors strengthening competence (*f* = 37)	The importance of consistent and uniform PC methods and practices (*f* = 17)
		Postgraduate education provided by the employer to strengthen competence (*f* = 14)
		The role of nursing directors enabling competence enhancement (*f* = 6)

### Health care professionals’ views of PC competency in nonspecialist settings

Building the foundations of one’s own competence included three categories. In the category *“The competence built up from work experience and support of a more experienced colleague,”* the participants highlighted that competence could grow through work experience or be lacking due to limited work experience. In the category *“Support from the specialist level of PC,”* respondents indicated that support from a specialist PC nurse or PC networks had strengthened their competence. In addition, as described by respondents in the category *“Education as the foundation of PC competence,”* both postgraduate and undergraduate education were also perceived as a basis of competence.

*Most of the know-how and “training” has come from learning from work situations. (P298)*

*The skills are based on nursing education and training as a practical nurse and have been supported by knowledge gained through experience and short refresher courses. (V80)*

Factors related to the work organization and connection to competence enhancement included two categories. As the respondents described in the category *“Employer related obstacles for achieving competencies,”* the lack of continuing education provided by the employer and the connection of rarely occurring PC encounters were perceived as a barrier to competence. On the other hand, health care professionals also identified “Employer-related factors strengthening competence,” such as the importance of consistent PC methods and practices as well as postgraduate education provided by the employer.

*In working life, despite my requests, I have not been able to participate in continuing education. (P270)*

*After completing the training, I felt I had the skills for palliative care. Now that I haven’t needed it in my work, things start to be forgotten. (P120)*

*Short educational repetitions have supported competence. (P80)*

### The development needs for continuous PC education in nonspecialist settings

A perceived level of PC competence and development needs included four categories. In the category *“Maintaining and developing one’s own competence,”* health care professionals agreed that recognizing the importance of PC competence and continuing education as well as the constant need to update one’s own skills played a key role in improving PC skills.

*However, it’s a very important topic from which it would be important to receive regular continuing education. (P604)*

*It would be good to be able to update the knowledge and skills I have learned in the past. (630)*

In the category *“Development needs of PC education,”* the health care professionals identified development needs in undergraduate nursing education and highlighted the importance of continuous and regular postgraduate education, as the following:

*When I graduated as a nurse… there was very little training on the (PC) subject. (P355)*

*Regularly (annually) recurring refresher training would be necessary for all staff in units with infrequent (monthly or less) attendance in hospice care. (P188)*

Furthermore, the respondents expressed their views on *“Development needs of PC competencies in different health care units,”* such as nursing homes, home care, and in-hospital care. They also described *“The perceived level of PC competence”* as variable, from insufficient to excellent.

*There are too few skilled workers in nursing homes. (P275)*

*There is high variation in competence between regions. (P594)*

A perceived need for continuing education in different PC competencies included eight categories. In the category *“Multiprofessional competencies”* participants shared the view that physician competence in PC should be developed, as well as competence in multiprofessional collaboration.

*The palliative care and hospice care skills of ward doctors are very modest, and there is no help from the senior doctor. The doctors do not listen to the trained nurses and do not consult the doctor in the palliative care unit. They should actively seek knowledge on the subject. (P8)*

*Skills should be developed across professions, between doctors and nurses and other professionals alike. (P592)*

The category *“Competence of health care professionals in pain management”* highlighted the need for continuing education for both nurses and physicians in that area. In addition to pain management, in the category *“Managing common symptoms,”*, respondents highlighted the management of other symptoms and the importance of both pharmacological and nonpharmacological interventions.

*In nursing, I have often encountered that a nurse who is more educated than me does not want to give the patient enough painkillers, even though the patient is in pain, or that the patient will have to wait too long for painkillers. (P55)*

*I feel that the doctors in my unit do not have enough knowledge in medication protocols in advance care planning, since I must “beg” for the patient to get it. (593)*

*There are still gaps in symptom management. (P300)*

A provision of spiritual, existential, and psychosocial support and multicultural needs were identified in the category *“Psychological, psychosocial and existential support in PC.”* In the category *“Respectful interaction and being with the patient,”* respondents described the importance of maintaining a calm and respectful atmosphere in challenging PC situations and of being empathetic with the patient. *“Encountering and supporting the closest ones”* covered respondents’ views on the skills needed by health care professionals to support, encounter, and guide those closest to the patient.

*Clear further training is needed in providing psychosocial support; how to face the existential suffering of a dying patient and how to support loved ones. (P461)*

*The most important thing would be to be present and listen. (P164)*

In the category *“Basic competencies in PC,”* participants identified educational needs in relation to different patient groups and in meeting patients’ basic needs. The category *“Advance care planning and documentation”* highlighted the shortcomings that were identified in the preparation of advance care plans and in the documentation of nursing in PC.

*The importance of basic care (mouth, constipation, urinary incontinence) should definitely be discussed and educated more. (P40)*

*We are not familiar with the concept of advance care planning. (P671)*

## Discussion

The purpose of this study was to examine the views of Finnish health care professionals regarding PC competence and the development needs of continuing education in nonspecialist settings. The respondents broadly described how PC competence was built up and highlighted that competence was often accumulated through practical work and learning from a more experienced colleague. The type of clinical experience and previous education in PC have been reported to have a crucial role when assessing the attitudes of nurses toward end-of-life care in a previous study.^30^

According to this study, specialized nurses and other colleagues with more additional training acted as support and strengthened PC competence. Earlier research has shown that providing informal education and guidance to colleagues are competencies that nurses specialized in PC or advanced nurse practitioners possess.^31^ Networking with PC experts was also emphasized as an important factor, as well as the support provided by PC centers. In recent years, a lot of development work has been done in Finland to promote PC and its accessibility. The establishment of palliative units at both university hospitals and smaller regional hospitals, as well as an expanding network of home hospitals and palliative consultation activities, have brought much-needed support to those working at nonspecialist levels of PC.^24^ Developing collaboration across different levels of PC and ensuring the consultation pathways throughout health care is one of the focal points globally.^9,32^

In this study, PC education within undergraduate degrees was addressed as very important but at the same time often quite insufficient. These results are in line with previous research emphasizing that PC education should be integrated more widely and more strongly into undergraduate nursing and medical studies.^33–36^ This study showed that it was strongly emphasized that one’s own professional skills and knowledge should be constantly updated. Continuing education should be regular and continuous, for in such contexts where PC is not daily or regularly repeated (nonspecialist levels of PC), acquired skills are easily forgotten and the health care professional begins to feel insecure. This paradox of nurses feeling unprepared to provide high-quality PC after graduation has been recognized in previous studies as well.^37,38^ In nonspecialist levels of PC, the employees recognized the imminent need for continuous education, but often felt that factors connected to the employer are an obstacle to competence development. Furthermore, the expensive cost of PC specialization studies impedes continuing education when the employer often does not want to contribute to the costs. Respectively, this study showed that if especially nursing supervisors supported attending continuous education, competencies will be preserved and developed. The importance of the support from an employing authority and nursing supervisors has been identified in earlier studies as well.^31,39,40^

Inequities in PC have been recognized and defined as health care priorities globally.^1,6–8^ Not only the availability of PC but also factors related to competencies and quality of care have been highlighted recently.^9,21,24,38–43^ Participants of this study expressed the perceived level of PC competence in numerous ways. The level of PC competence was seen as very variable, both at the individual and organizational level. Overall, the level of competence was described as varying from insufficient to excellent.

In this study, health care professionals pointed out the need to improve PC competencies especially in different nonspecialist levels of PC health care environments. The lack of competence was described as connected to, for example, delays in decision-making and advance care planning protocols and insufficient symptom treatment, or generally a weaker implementation of comprehensive, and person-centered care. In addition, the lack of PC competence leads sometimes to unnecessary last-minute hospital transfers. Similar issues have been highlighted in previous studies as well.^44–51^

Based on this study, PC competence development should apply to all health care professional groups. Most of the respondents to this survey were nurses (Table 1), and according to their views, physicians should have more expertise in identifying the need for PC, defining goals of care and advance care planning, as well as in pain management and pharmacological interventions. These competencies required of physicians working within PC have been described in previous research.^36^ The importance of multiprofessional competence and cooperation was considered important and should be included in PC continuing education, as has also been emphasized in earlier research.^41,52^

The views of the participants in this study about the key content areas of PC continuing education were consistent with previous research data.^11,20,21,31,33–39^ Preferred training methods were identified in this study, such as including simulation training or various scenario videos as part of continuing education. Easier accessibility directly from the workplace was seen as an advantage of online training, but there was also a need for educational events in person. In addition, the training skills of teachers and experts should be improved. These themes have emerged also in earlier studies regarding PC education on both the undergraduate and postgraduate levels.^23,42,53^

### Strengths and limitations of the study

The trustworthiness of the study was strengthened by choosing a research method that was suitable for the purpose of the study. In addition, the study sample represented widely diverse nonspecialist PC settings of care nationally. Therefore, the data can be considered as comprehensively representing the phenomenon under study. Moreover, data saturation was achieved during the analysis.^27^ An example of the analysis process (Table 2) and the tables of categories identified through content analysis (Tables 3 and 4) strengthen the dependability, as well as the citations from the data which strengthen the authenticity.^26^

**Table 4.** The Development Needs for Continuous PC Education in Nonspecialist Settings

Main category	Category	Subcategory
A perceived level of PC competence and development needs (*f* = 303)	Maintaining and developing one’s own competence (*f* = 133)	Identifying the importance of PC competencies and continuing education (*f* = 47)
		The constant need to update one’s own competencies (*f* = 45)
		Identifying the need for continuing education (*f* = 41)
	Development needs of PC education (*f* = 69)	The development needs of undergraduate nursing education (*f* = 19)
		The importance of continuous and regular postgraduate education (*f* = 18)
		The development needs of training implementation and methods in postgraduate education (*f* = 15)
	Development needs of PC competencies in different health care units (*f* = 55)	The need to develop PC competencies in homes for the aged (*f* = 17)
		The need to develop PC competencies in home care (*f* = 15)
		The need to develop PC competencies in hospital care (*f* = 10)
		The need to develop PC competencies in basic health care (*f* = 6)
		The need to develop PC competencies in the emergency units (*f* = 3)
		The need to develop PC competencies in basic health care centers and hospitals (*f* = 2)
		Unnecessary patient transfers due to a lack of PC skills (*f* = 2)
	Perceived level of PC competence (*f* = 46)	Insufficient level of competence (*f* = 13)
		Variability in the level of competence (*f* = 12)
		Sufficient level of competence (*f* = 6)
		Good level of competence (*f* = 6)
		Excellent level of competence (*f* = 6)
		Identified competence development (*f* = 3)
A perceived need for continuing education in different PC competencies (*f* = 243)	Multiprofessional competencies (*f* = 44)	Perceived need to develop the PC competencies of doctors (*f* = 35)
		Competence in multiprofessional collaboration (*f* = 9)
	Competence of health care professionals in pain management (*f* = 42)	Competence of nurses in pain management (*f* = 39) Competence of doctors in pain management (*f* = 3)
	Psychological, psychosocial and existential support in PC (*f* = 37)	Provision of spiritual and existential support (*f* = 10) Provision of psychosocial support (*f* = 8)
		Provision of psychological support (*f* = 8)
		Multicultural needs (*f* = 6)
		Supporting the patient (*f* = 4)
		Taking care of the quality of life (*f* = 1)
	Respectful interaction and being present with the patient (*f* = 34)	Maintaining a calm and respectful atmosphere in challenging PC situations (*f* = 14)
		Empathetic and respectful encounter (*f* = 11)
		Genuine presence (*f* = 5)
		Listening to the patient (*f* = 4)
	Encountering and supporting the closest ones (*f* = 31)	Supporting the patient’s closest ones throughout the trajectory of PC (*f* = 15)
		Encountering the patient’s closest ones (*f* = 9)
		Guidance of the patient’s closest ones (*f* = 5)
		Listening to the patient’s closest ones (*f* = 2)
	Managing common symptoms (*f* = 23)	Pharmacological interventions in symptom management (*f* = 10)
		Management of symptoms (*f* = 8)
		Nonpharmacological interventions in symptom management (*f* = 5)
	Basic competencies in PC (*f* = 17)	Educational needs related to different patient groups (*f* = 10)
		Taking care of patient’s basic needs (*f* = 7)
	Advance care planning and documentation (*f* = 15)	Advance care planning (*f* = 8) Palliative nursing care documentation (*f* = 5)
		Assessment of the need for care (*f* = 2)

Nevertheless, the research also included limitations possibly weakening the trustworthiness of the study. Only some of the participants who answered the entire survey answered the open question. As the survey was answered anonymously, it is impossible to know the reasons why certain respondents refused to answer this question. In addition, asking any further questions was not possible, as well as returning the results for the participants to review and to provide feedback.

## Conclusions

This study provides evidence, that there is variety in the PC competency in Finnish nonspecialist settings. Performing equally high-quality PC at any nonspecialist level requires multiprofessional and comprehensive competence. The findings highlight the importance of continuing education, as the health care professionals reported feelings of unpreparedness and experiences of insufficient PC education and competencies. Furthermore, in this study, there were preferred continuing education methods and PC contents. Based on the results of this study, we suggest that health care organizations and employers commit to supporting competence development and continuous education in PC.
