# Supplementary material for: Levels of Competence and Need for Continuing Education in Nonspecialist Palliative Care Settings—A Qualitative Study of Views from Finnish Health Care Professionals
Source: Palliat Med Rep. 2024 Dec 23;5(1):553–62. doi: 10.1089/pmr.2024.0060 (PMC11848055; doi:10.1089/pmr.2024.0060)
Supplement: Supplementary Data [file pmr.2024.0060_supp_datas1.docx]

**Supplementary 1. The statements of competency, clinical nursing staff and supervisors**

**Theme 1. Client-centered care and advance directives**.

| **Statement** | **Completely disagree** | **Partly disagree** | **Don’t agree or disagree** | **Partly agree** | **Completely agree** |  | **Can’t say** |
| --- | --- | --- | --- | --- | --- | --- | --- |
| **Clinical nursing staff: Theme 1. Client-centered care and limitations of treatment** | | | | | | | |
| I understand the significance of the living will in palliative care. |  |  |  |  |  |  |  |
| I understand the significance of limitations of treatment in palliative care. |  |  |  |  |  |  |  |
| I know how to guide the patient in matters related to living wills. |  |  |  |  |  |  |  |
| I know how to assess and monitor any necessary changes related to a patient’s living will. |  |  |  |  |  |  |  |
| Within the limitations of my role, I know how to give the patient enough information about their medical condition. |  |  |  |  |  |  |  |
| I know the policies related to the documentation of living wills and limitations of treatment. |  |  |  |  |  |  |  |
|  | | | | | | | |
| **Supervisors: Theme 1. Client-centered care and advance directives** | | | | | | | |
| In my work unit, staff members understand the significance of the living will in palliative care. |  |  |  |  |  |  |  |
| In my work unit, staff members understand the significance of limitations of treatment in palliative care. |  |  |  |  |  |  |  |
| In my work unit, staff members know how to guide the patient in matters related to living wills. |  |  |  |  |  |  |  |
| In my work unit, staff members know how to assess and monitor any necessary changes related to a patient’s living will. |  |  |  |  |  |  |  |
| In my work unit, staff members know how to give the patient enough information about their medical condition. |  |  |  |  |  |  |  |
| In my work unit, staff members know the policies related to the documentation of living wills and limitations of treatment. |  |  |  |  |  |  |  |

**Theme 2. Communication with patients’ closest ones.**

| **Statement** | **Completely disagree** | **Partly disagree** | **Don’t agree or disagree** | **Partly agree** | **Completely agree** |  | **Can’t say** |
| --- | --- | --- | --- | --- | --- | --- | --- |
| **Staff: Theme 2. Communication with patients’ closest ones** | | | | | | | |
| I know matters related to guardianship and power of attorney. |  |  |  |  |  |  |  |
| I understand the need for support from closest ones and know how to respond to it |  |  |  |  |  |  |  |
| I know how to guide and give necessary information to closest ones. |  |  |  |  |  |  |  |
|  | | | | | | | |
| **Supervisors: Theme 2. Communication with patients’ closest ones** | | | | | | | |
| In my work unit, staff members know the matters related to guardianship and power of attorney. |  |  |  |  |  |  |  |
| In my work unit, staff members understand the need for support from closest ones and know how to respond to it. |  |  |  |  |  |  |  |
| In my work unit, staff members know how to guide and give necessary information to closest ones. |  |  |  |  |  |  |  |

**Theme 3. Advance care planning**

| **Statement** | **Completely disagree** | **Partly disagree** | **Don’t agree or disagree** | **Partly agree** | **Completely agree** |  | **Can’t say** |
| --- | --- | --- | --- | --- | --- | --- | --- |
| **Clinical nursing staff: Theme 3. Advance care planning** | | | | | | | |
| I understand what an advance care plan (ACP) for end-of-life care means |  |  |  |  |  |  |  |
| I know what an ACP should include. |  |  |  |  |  |  |  |
| I know how to provide care that is concordant with an ACP. |  |  |  |  |  |  |  |
| I know how to, if necessary, initiate discussions about the need to create an ACP for end-of-life care. |  |  |  |  |  |  |  |
| I know how to assess a need for updating ACP. |  |  |  |  |  |  |  |
| I know how to start an update process on a patient’s ACP when necessary. |  |  |  |  |  |  |  |
| I am aware of the documentation practices regarding palliative care and end-of-life ACP. |  |  |  |  |  |  |  |
|  | | | | | | | |
| **Supervisors: Theme 3. Advance care planning** | | | | | | | |
| In my work unit, staff members understand what an advance care plan (ACP) for end-of-life care means. |  |  |  |  |  |  |  |
| In my work unit, staff members know what an ACP should include. |  |  |  |  |  |  |  |
| In my work unit, staff members know how to provide care that is concordant with an ACP. |  |  |  |  |  |  |  |
| In my work unit, staff members know how to, if necessary, initiate discussions about the need to create an ACP for end-of-life care. |  |  |  |  |  |  |  |
| In my work unit, staff members know how to assess a need for updating ACP. |  |  |  |  |  |  |  |
| In my work unit, staff members know how to start an update process on a patient’s ACP when necessary. |  |  |  |  |  |  |  |
| In my work unit staff members are aware of the documentation practices regarding palliative care and end-of-life ACP. |  |  |  |  |  |  |  |

**Theme 4. Ensuring competence**

| **Statement** | **Completely disagree** | **Partly disagree** | **Don’t agree or disagree** | **Partly agree** | **Completely agree** |  | **Can’t say** |
| --- | --- | --- | --- | --- | --- | --- | --- |
| **Clinical nursing staff: Theme 4. Ensuring competence** | | | | | | | |
| I know how to evaluate and describe pain. |  |  |  |  |  |  |  |
| I know how to treat pain. |  |  |  |  |  |  |  |
| I know how to evaluate symptoms related to dry mouth. |  |  |  |  |  |  |  |
| I know how to treat symptoms related to dry mouth. |  |  |  |  |  |  |  |
| I know how to evaluate nausea and vomiting. |  |  |  |  |  |  |  |
| I know how to treat nausea and vomiting. |  |  |  |  |  |  |  |
| I know how to assess constipation. |  |  |  |  |  |  |  |
| I know how to treat constipation. |  |  |  |  |  |  |  |
| I know how to assess dyspnea. |  |  |  |  |  |  |  |
| I know how to treat dyspnea. |  |  |  |  |  |  |  |
| I know how to prevent pressure ulcers. |  |  |  |  |  |  |  |
| I know how to assess pressure ulcers. |  |  |  |  |  |  |  |
| I know how to treat pressure ulcers. |  |  |  |  |  |  |  |
| I know how to assess patients for depression. |  |  |  |  |  |  |  |
| I know how to treat depression. |  |  |  |  |  |  |  |
| I know how to assess insomnia. |  |  |  |  |  |  |  |
| I know how to treat insomnia. |  |  |  |  |  |  |  |
| I know how to assess seizures. |  |  |  |  |  |  |  |
| I know how to treat seizures. |  |  |  |  |  |  |  |
| I know how to assess fatigue. |  |  |  |  |  |  |  |
| I know how to treat fatigue. |  |  |  |  |  |  |  |
| I know how to assess anxiety. |  |  |  |  |  |  |  |
| I know how to treat anxiety. |  |  |  |  |  |  |  |
| I know how to evaluate existential suffering. |  |  |  |  |  |  |  |
| I know how to treat existential suffering. |  |  |  |  |  |  |  |
| I know how to consider their life philosophy, religion, beliefs, and/or cultural background while caring for a patient/client. |  |  |  |  |  |  |  |
| I know how to recognize the signs of approaching death. |  |  |  |  |  |  |  |
| I know how to implement palliative care and end-of-life symptom management using medication administered orally, as tablets, capsules, solutions, eye and ear drops, ointments, patches, and suppositories. |  |  |  |  |  |  |  |
| I know how to administer medications used in palliative care using subcutaneous injections or infusions. |  |  |  |  |  |  |  |
| I know how to administer medications used in palliative care using intravenous injections or infusions. |  |  |  |  |  |  |  |

| **Statement** | **Completely disagree** | **Partly disagree** | **Don’t agree or disagree** | **Partly agree** | **Completely agree** |  | **Can’t say** |
| --- | --- | --- | --- | --- | --- | --- | --- |
| **Supervisors: Theme 4. Ensuring competence** | | | | | | | |
| In my work unit, the staff members know how to evaluate and describe pain. |  |  |  |  |  |  |  |
| In my work unit, the staff members know how to treat pain. |  |  |  |  |  |  |  |
| In my work unit, the staff members know how to evaluate symptoms related to dry mouth. |  |  |  |  |  |  |  |
| In my work unit, the staff members know how to treat symptoms related to dry mouth. |  |  |  |  |  |  |  |
| In my work unit, the staff members know how to evaluate nausea and vomiting. |  |  |  |  |  |  |  |
| In my work unit, the staff members know how to treat nausea and vomiting. |  |  |  |  |  |  |  |
| In my work unit, the staff members know how to assess constipation. |  |  |  |  |  |  |  |
| In my work unit, the staff members know how to treat constipation. |  |  |  |  |  |  |  |
| In my work unit, the staff members know how to assess dyspnea. |  |  |  |  |  |  |  |
| In my work unit, the staff members know how to treat dyspnea. |  |  |  |  |  |  |  |
| In my work unit, the staff members know how to prevent pressure ulcers. |  |  |  |  |  |  |  |
| In my work unit, the staff members know how to assess pressure ulcers. |  |  |  |  |  |  |  |
| In my work unit, the staff members know how to treat pressure ulcers. |  |  |  |  |  |  |  |
| In my work unit, the staff members know how to assess patients for depression. |  |  |  |  |  |  |  |
| In my work unit, the staff members know how to treat depression. |  |  |  |  |  |  |  |
| In my work unit, the staff members know how to assess insomnia. |  |  |  |  |  |  |  |
| In my work unit, the staff members know how to treat insomnia. |  |  |  |  |  |  |  |
| In my work unit, the staff members know how to assess seizures. |  |  |  |  |  |  |  |
| In my work unit, the staff members know how to treat seizures. |  |  |  |  |  |  |  |
| In my work unit, the staff members know how to assess fatigue. |  |  |  |  |  |  |  |
| In my work unit, the staff members know how to treat fatigue. |  |  |  |  |  |  |  |
| In my work unit, the staff members know how to assess anxiety. |  |  |  |  |  |  |  |
| In my work unit, the staff members know how to treat anxiety. |  |  |  |  |  |  |  |
| In my work unit, the staff members know how to evaluate existential suffering. |  |  |  |  |  |  |  |
| In my work unit, the staff members know how to treat existential suffering. |  |  |  |  |  |  |  |
| In my work unit, the staff members know how to consider a patient's life philosophy, religion, beliefs, and/or cultural background while caring for them. |  |  |  |  |  |  |  |
| In my work unit, the staff members know how to recognize the signs of approaching death. |  |  |  |  |  |  |  |
| In my work unit, the staff members know how to implement palliative care and end-of-life symptom management using medication administered orally, as tablets, capsules, solutions, eye and ear drops, ointments, patches, and suppositories. |  |  |  |  |  |  |  |
| In my work unit, the staff members know how to administer medications used in palliative care using subcutaneous injections or infusions. |  |  |  |  |  |  |  |
| In my work unit, the staff members know how to administer medications used in palliative care using intravenous injections or infusions. |  |  |  |  |  |  |  |

**Theme 5. Care pathways and availability of expertise**

| **Statement** | **Completely disagree** | **Partly disagree** | **Don’t agree or disagreedisagree** | **Partly agree** | **Completely agree** |  | **Can’t say** |
| --- | --- | --- | --- | --- | --- | --- | --- |
| **Clinical nursing staff: Theme 5. Care pathways and availability of expertise** | | | | | | | |
| I know the palliative care consultation practices within my organization. |  |  |  |  |  |  |  |
| When needed, I know how to consult other professionals and services related to my patients’ care. |  |  |  |  |  |  |  |
| I know how to evaluate my patients’ need for psychosocial support and organize it when needed. |  |  |  |  |  |  |  |
| I know how to evaluate my patients’ need for existential/spiritual support and organize it when needed. |  |  |  |  |  |  |  |
|  | | | | | | | |
| **Supervisors: Theme 5. Care pathways and availability of expertise** | | | | | | | |
| In my work unit, the staff members know the palliative care consultation practices within my organization. |  |  |  |  |  |  |  |
| In my work unit, the staff members know how to consult other professionals and services related to my patients’ care. |  |  |  |  |  |  |  |
| In my work unit, the staff members know how to evaluate my patients’ need for psychosocial support and organize it when needed. |  |  |  |  |  |  |  |
| In my work unit, the staff members know how to evaluate patients’ needs for existential/spiritual support and organize it when needed. |  |  |  |  |  |  |  |

**Theme 6: Structural factors and medical devices**

| **Statement** | **Completely disagree** | **Partly disagree** | **Don’t agree or disagree** | **Partly agree** | **Completely agree** |  | **Can’t say** |
| --- | --- | --- | --- | --- | --- | --- | --- |
| **Clinical nursing staff: Theme 6: Structural factors and medical devices** | | | | | | | |
| I know how to recognize the need for privacy for a patient in end-of-life care. |  |  |  |  |  |  |  |
| I know how to identify and facilitate the involvement of the closest ones in the end-of-life care of a patient. |  |  |  |  |  |  |  |
| I know how to identify the individual needs and wishes of a patient in palliative care (e.g., desire to go outside while in bed). |  |  |  |  |  |  |  |
| I know how to use assistive technology in caring for a dying person. |  |  |  |  |  |  |  |
|  | | | | | | | |
| **Supervisors: Theme 6: Structural factors and medical devices** | | | | | | | |
| In my work unit, the staff members know how to recognize the need for privacy for a patient in end-of-life care. |  |  |  |  |  |  |  |
| In my work unit, the staff members know how to identify and facilitate the involvement of the closest ones in the end-of-life care of a patient. |  |  |  |  |  |  |  |
| In my work unit, the staff members know how to identify the individual needs and wishes of a patient in palliative care (e.g., desire to go outside while in bed). |  |  |  |  |  |  |  |
| In my work unit, the staff members know how to use assistive technology in caring for a dying person. |  |  |  |  |  |  |  |

**Theme 7: Bereavement and postmortem care**

| **Statement** | **Completely disagree** | **Partly disagree** | **Don’t agree or disagree** | **Partly agree** | **Completely agree** |  | **Can’t say** |
| --- | --- | --- | --- | --- | --- | --- | --- |
| **Clinical nursing staff:** **Theme 7: Bereavement and postmortem care** | | | | | | | |
| I know what to do when the patient has died. |  |  |  |  |  |  |  |
| I know how to discuss death and dying with patients’ closest ones. |  |  |  |  |  |  |  |
| I know how to comfort and support those who have lost their loved ones. |  |  |  |  |  |  |  |
| I know how to support closest ones taking part in the respectful care for the deceased. |  |  |  |  |  |  |  |
| I know how to respectfully care for the deceased. |  |  |  |  |  |  |  |
| I know what to do if the deceased has a drain, a central venous catheter, or a pacemaker. |  |  |  |  |  |  |  |
| I know how to consider the individual cultural and persuasion related needs of the deceased and their closest ones. |  |  |  |  |  |  |  |
| I know how to guide the closest ones regarding documents related to the death of their loved one. |  |  |  |  |  |  |  |
| I know how to provide guidance as needed for the closest ones regarding support in bereavement. |  |  |  |  |  |  |  |
| I know how to provide appropriate psychosocial and/or emotional support according to the needs of the closest ones. |  |  |  |  |  |  |  |
|  | | | | | | | |
| **Supervisors:** **Theme 7: Bereavement and postmortem care** | | | | | | | |
| In my work unit, staff members know what to do when the patient has died. |  |  |  |  |  |  |  |
| In my work unit, staff members know how to discuss death and dying with the patients’ closest ones. |  |  |  |  |  |  |  |
| In my work unit, staff members know how to comfort and support those who have lost their loved ones. |  |  |  |  |  |  |  |
| In my work unit, staff members know how to support the closest ones taking part in the respectful care for the deceased. |  |  |  |  |  |  |  |
| In my work unit, staff members know how to respectfully care for the deceased. |  |  |  |  |  |  |  |
| In my work unit, staff members know what to do if the deceased has a drain, a central venous catheter, or a pacemaker. |  |  |  |  |  |  |  |
| In my work unit, staff members know how to consider the individual cultural and persuasion related needs of the deceased and their closest ones. |  |  |  |  |  |  |  |
| In my work unit, staff members know how to guide the closest ones regarding documents related to the death of their loved one. |  |  |  |  |  |  |  |
| In my work unit, staff members know how to provide guidance as needed for the closest ones regarding support in bereavement. |  |  |  |  |  |  |  |
| In my work unit, staff members know how to provide appropriate psychosocial and/or emotional support according to the needs of the closest ones. |  |  |  |  |  |  |  |
